# Low Grade Appendiceal Mucinous Neoplasm – A Case Series A Silent Threat with Deadly Consequences 

**DOI:** 10.30699/ijp.2025.2055124.3421

**Published:** 2025-07-01

**Authors:** S.J. John Joseph Hemnath, Shanmugapriya M, Suresh R, Madhumita Paleri, Lokeshwari V, Eswari V

**Affiliations:** 1 *Meenakshi Medical College Hospital and Research Institute, Kanchipuram, Tamilnadu, India *; 2 *Meenakshi Academy of Higher Education and Research, Chennai, India*

**Keywords:** Appendiceal Neoplasms, Pseudomyxoma Peritonei, Immunohistochemistry

## Abstract

**Background & Objective:**

Our study was mainly aimed at identifying the cause, clinical course of the disease, its most dreaded complication pseudomyxoma peritonei and spread to adjacent structures. We have used IHC techniques to know the origin of the tumor where both appendix and ovary were involved.

**Case Presentation:**

We have listed out and discussed in detail 5 cases, each of which have a different clinical course and variation in staging, grading and prognosis. One of the cases had mucin deposit in the ovary for which IHC was done to confirm the origin of the tumor. Some of the findings were incidental and in others presence of mucin content in the appendix lumen raised clinical suspicion of spread to peritoneum and adjacent structures. Grading and staging is of prime importance as it determines the prognosis and management of the patient respectively.

**Conclusion:**

A ruptured or perforated appendix must warrant for an immediate suspicion of pseudomyxoma peritonei which when untreated can lead to mucinous neoplasms in adjacent organs like ovary and colon. Pseudomyxoma Peritonei (PMP) with simultaneous appendix and ovarian neoplasm should be treated as primary appendiceal tumour. Grading and staging of mucinous neoplasm of appendix and Pseudomyxoma Peritoneii needs a unified approach for standardized diagnostic reporting.

## Introduction

Low-grade appendiceal mucinous neoplasm (LAMN) is a rare type of appendiceal tumor, accounting for approximately 0.2–0.3% of all appendectomy specimens (1). It is often diagnosed incidentally but can lead to a potentially fatal complication known as pseudomyxoma peritonei (PMP), characterized by the accumulation of mucinous material in the peritoneal cavity.

The Peritoneal Surface Oncology Group International (PSOGI) has established diagnostic criteria for LAMN, while the 8th edition of the American Joint Committee on Cancer (AJCC) staging system and the 5th edition of the World Health Organization (WHO) classification provide guidelines for grading and staging these tumors.

In this report, we present five cases of LAMN, one of which exhibited mucinous deposits in the ovary. Immunohistochemistry (IHC) was performed in this case to determine the primary origin of the tumor.

## Cases description

### Study Design and Duration: 

This is a combined retrospective and prospective case series conducted over a period of three years (2021–2024) at Meenakshi Medical College Hospital and Research Institute, Kanchipuram, Tamil Nadu, India. During this period, no primary appendiceal cancers (other than LAMN) were identified, while 14 ovarian tumors were reported. Out of these, five cases of low-grade appendiceal mucinous neoplasm (LAMN) were identified and included in this series. One case involved both the appendix and the ovary, with the appendiceal origin confirmed by immunohistochemistry.

### Case Series

**Case 1**

**Fig 1:**
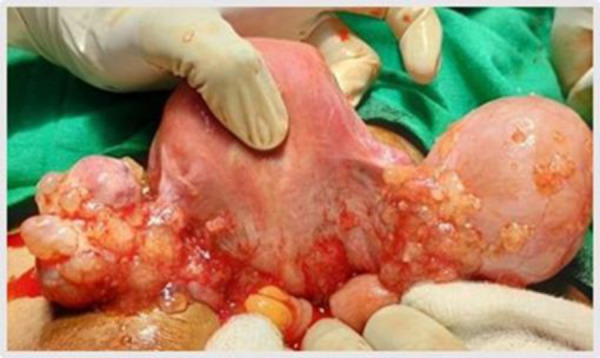
Intraoperative picture of Case 1 showing numerous mucin filled vesicles on bilateral ovarian surface

A 40-year-old female presented with intermittent lower abdominal pain for one year, insidious in onset and progressively worsening, along with heavy menstrual bleeding. Pelvic ultrasound revealed a right ovarian hemorrhagic cyst (4.9 × 2.9 cm) and a complex left ovarian cyst (4 × 2.8 cm) with minimal solid components. A total abdominal hysterectomy with bilateral salpingo-oophorectomy and appendectomy was planned. Intraoperative findings included multiple fluid-filled vesicles over the posterior omentum, peritoneum, bowel, uterus, and bilateral ovaries (Figure 1). The serum CA-125 was 143 U/mL.

**Fig 2:**
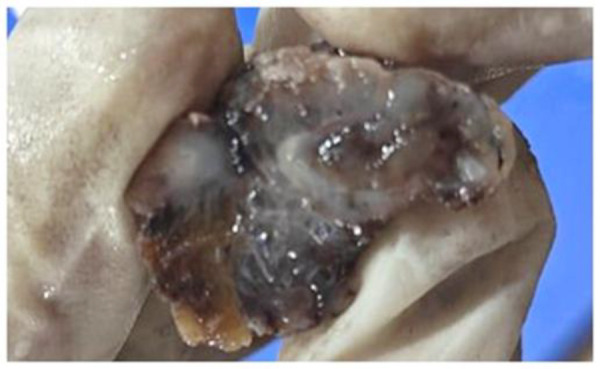
shows cut surface of appendix with mucinous material (Case 1).

**Fig. 3:**
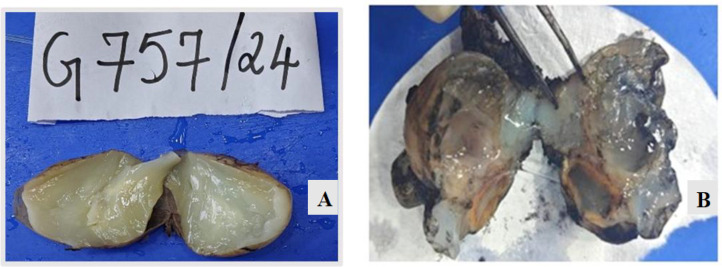
**A,** **B**. shows cut surface of right and left ovary with abundant gelatinous material (Case 1).

Grossly, the right ovary (6.8 × 5.5 × 3.5 cm) contained a uniloculated cyst filled with gelatinous material. No solid areas or papillary excrescences were noted. The left ovary (6.3 × 3.9 × 3.0 cm) showed multiple gelatin-filled cysts (Figure 3A, 3B). The appendix (4 × 2.6 × 1.5 cm) exhibited a mucin-filled lumen and a mass at the base (2.5 × 2.0 × 1.5 cm) (Figure 2).

**Fig 4:**
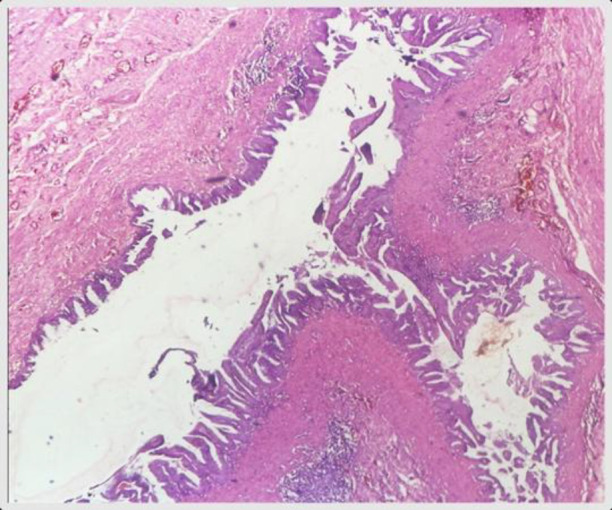
H & E (10X) shows neoplasm arising in villous architecture from appendiceal lumen (case 1)

**Fig 5:**
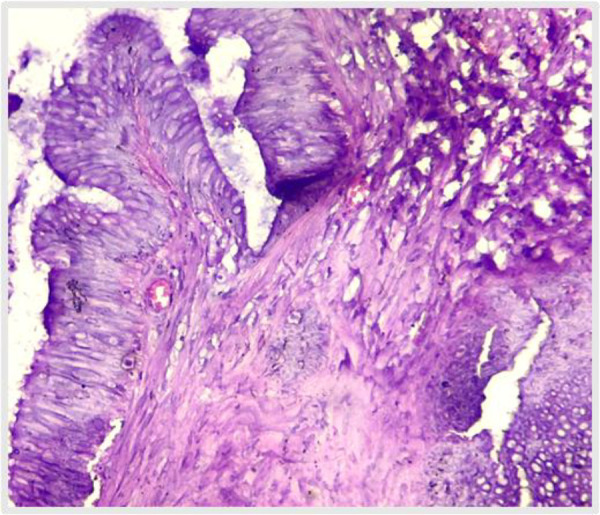
H & E (40X) shows villi are lined by columnar epithelium with abundant mucin and elongated nucleus (case 1).

**Fig. 6:**
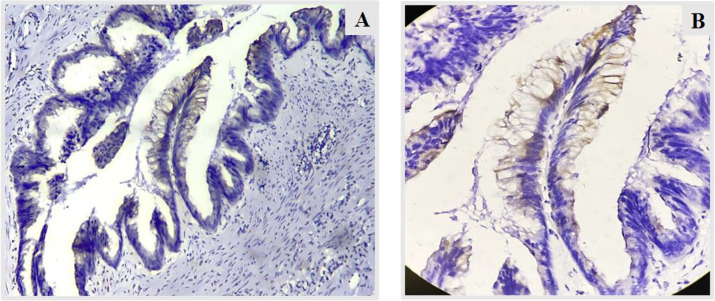
A,B – CK 20 (10X and 40X) – staining of the mucinous appendiceal lining cells with CK20 indicating that the tumor has originated from appendix (Case 1).

**Fig 7:**
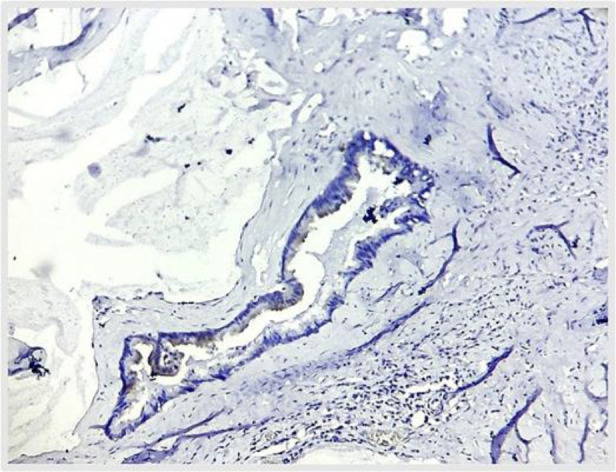
CK20 (10X) – shows positive staining of appendiceal lumen.

**Fig 8:**
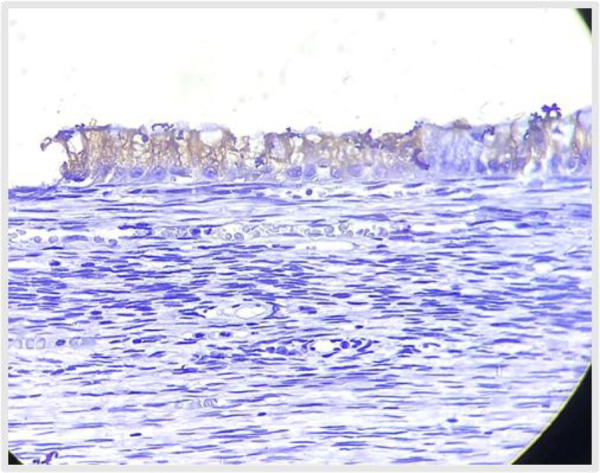
CK20 (40X) – shows positive staining of ovarian cyst wall lining

**Fig. 9:**
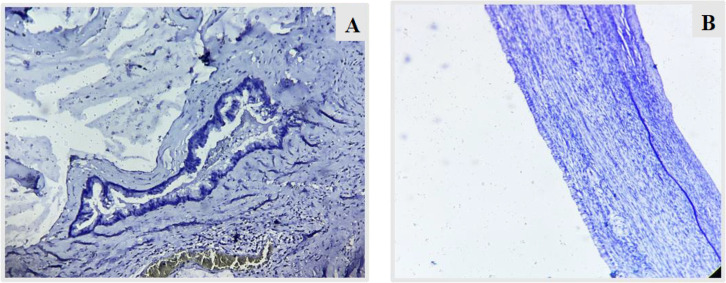
A, B – CK 7 (10X) – shows no staining in the appendiceal lumen and ovarian cyst wall lining as well, indicating that tumor has not originated from the ovaries (Case 1).

Microscopically, both ovaries revealed multiloculated cysts lined by pseudostratified mucinous columnar epithelium with goblet cells, mucin pools, and focal papillary structures. The appendiceal mass showed villous architecture lined by columnar mucinous epithelium with mucinous glands and pools dissecting into the wall, without lymphovascular invasion. Tumor deposits were seen on the serosal surface and resection margin. The lesion was diagnosed as LAMN (G1), pathologically staged as pT4b Nx M1b. Immunohistochemistry showed CK20 positivity and CK7 negativity, confirming appendiceal origin (Figures 4–9).

**Case 2**

A 60-year-old female presented with abdominal pain, nausea, vomiting, and anorexia for five days. Examination revealed right iliac fossa tenderness and a 2 × 2 cm mass. Ultrasound showed a heteroechoic collection (6.6 × 5.7 × 5.2 cm) near the appendix with psoas involvement. Appendectomy was performed.

Grossly, the appendix measured 4 cm and had a perforated tip with mucinous material in the lumen.

Microscopically, the perforation site showed flattened/pseudostratified epithelium with submucosal fibrosis, diverticulum-like growth, and acellular mucin dissection in the wall. No epithelial cells were seen in the mucin. The peritoneal biopsy showed fibrocollagenous tissue with acellular mucin and inflammation. A diagnosis of LAMN was made.

**Case 3**

A 26-year-old female presented with abdominal pain for two months and fever for four days. Three months earlier, she had undergone peritoneal lavage and appendectomy, with histology revealing LAMN (pT3 Nx). Upon recurrence of symptoms, CT scan revealed intestinal obstruction and mucinous deposits on the ileum with lymphadenopathy. She underwent right hemicolectomy with ileotransverse anastomosis.

Grossly, the right hemicolectomy specimen measured 35 cm, with a grey-black lesion (2 × 1.8 × 0.4 cm) in the terminal ileum. Mucinous deposits (2.5 × 1.8 × 0.8 cm) were also sampled.

Microscopically, mucosal ulceration, lymphoid follicular hyperplasia, and inflammation were noted in the ileum. Mucinous deposits contained acellular mucin with inflammatory infiltrates. No malignant cells were observed. The surgery was prophylactic to prevent disease spread, and absence of malignancy in deposits suggested a better prognosis.

**Case 4**

A 68-year-old female presented to the emergency department with diffuse abdominal pain, distension, bilious vomiting, and obstipation. History revealed similar intermittent pain over the past year. Examination showed distension, guarding, rigidity, and hyperperistalsis. CECT showed dilated jejunal and ileal loops with fluid, suggesting subacute obstruction. A mucocele of the appendix and free fluid in the right iliac fossa were also noted.

She underwent exploratory laparotomy. Intraoperatively, mucinous material was seen in the right iliac fossa, and the appendix was dilated and inflamed.

Grossly, the appendix (4.7 × 3 × 2 cm) had mucinous material within a thickened wall (0.2–0.5 cm). The peritoneal biopsy measured 5.2 × 2.5 × 0.5 cm.

**Fig. 10:**
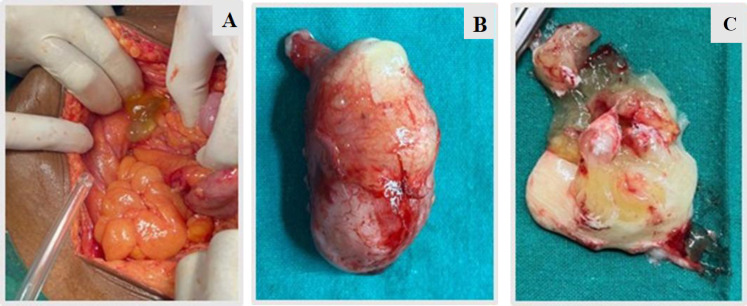
(A) Intraoperative picture showing mucinous substance in right iliac fossa region, (B) shows gross appearance of mucus filled appendix, (C) shows mucinous contents which were present inside the appendix.

**Figure 11:**
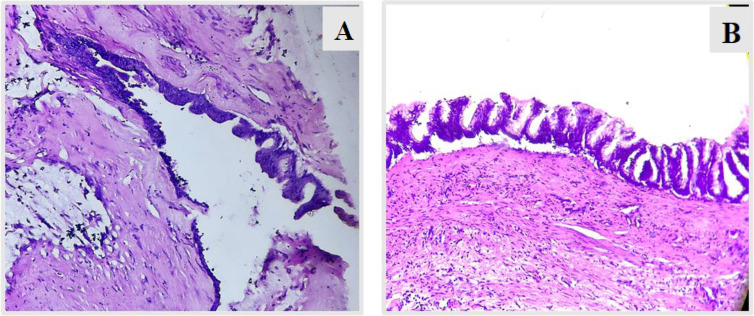
. **(A)** H&E (10X) shows distal appendix lined by mucinous columnar epithelium in villous pattern, **(B)** H&E (40X) shows tumor cells with moderate mucinous cytoplasm, hyperchromatic nuclei and mild nuclear atypia.

Microscopically, the appendix showed mucinous epithelium with mild atypia, villous pattern, and mucin pools. Acellular mucin dissected through the muscularis into the serosa. Peritoneal tissue showed tumor cells with mucinous deposits and calcification. The diagnosis was LAMN (G1), staged as pT4a N0 M1b (CAP protocol) (Figures 10–11).

**Case 5**

A 35-year-old male presented with right iliac fossa pain, vomiting, and tenderness. Laparoscopic appendectomy was performed.

Grossly, the appendix measured 5.5 cm and showed a dilated lumen filled with mucin.

Microscopically, the appendix was lined by mucinous columnar epithelium with apical mucin, oval nuclei, and fine chromatin. Focal papillary projections were seen. The lumen contained abundant mucin. A diagnosis of LAMN was made.

The salient features of all five LAMN cases are summarized in Table 1, detailing demographics, key histologic findings, tumor grade, pathological staging, and clinical outcomes. Presentations ranged from incidental discovery to complications like pseudomyxoma peritonei and bowel obstruction. While all tumors were low grade (G1), staging varied from confined to the appendix (pTis) to advanced disease with peritoneal spread (pT4b). This case series highlights the clinical and pathological heterogeneity of LAMN and emphasizes the importance of early diagnosis and accurate staging to guide treatment and prognosis. 

## Discussion

Low-grade appendiceal mucinous neoplasm (LAMN) is a rare but clinically significant entity, accounting for 0.2–0.3% of all appendiceal specimens (1). The main clinical concern with LAMN is its potential to cause pseudomyxoma peritonei (PMP), a life-threatening complication resulting from mucinous dissemination within the peritoneal cavity (1). The classification and staging of LAMN have evolved, with the Peritoneal Surface Oncology Group International (PSOGI) and the AJCC 8th edition/WHO 5th edition providing standardized criteria. The common tumors of appendix are of epithelial and mesenchymal type. The mucinous neoplasms of appendix are usually of low grade which can generate abundant mucin that accumulates in the peritoneal cavity. In order to classify and grade LAMN we have to gross and submit the entire appendix to be processed. In 2016, PSOGI outlined the classification and definition for LAMN. Later AJCC along with WHO 5^th^ edition has helped in grading and consisting of the different terminologies used for mucinous neoplasms of appendix. PSOGI classified primary appendiceal tumors into invasive and non-invasive types. LAMN is classified under non-invasive type. According to PSOGI LAMN is defined by the following: Low-grade cytology and any of the following: • Loss of muscularis mucosae • Fibrosis of submucosa • Undulating or flattened epithelial growth • “Pushing invasion” (expansile or diverticulum like growth) • Dissection of acellular mucin in the wall • Mucin and/or neoplastic cells outside of the appendix. New category which was introduced is high grade appendiceal mucinous neoplasm (HAMN) high-grade cytology but with the neoplasm confined to the appendix without invasion. AJCC staged the LAMN as Tis - confined to the muscularis propria. Mucin or mucinous epithelium may extend into the muscularis propria. LAMNs often have different degrees of appendiceal wall fibrosis. Therefore, pT1 and pT2 staging in colorectal TNM staging is unsuitable for LAMNs. Literature evidence indicates that patients with pTis(LAMN) do not develop tumor recurrence and are essentially cured by appendectomy (1,2,5,7,9,15). T3 extends through the muscularis propria into the subserosa ormesoappendix. T4a penetrates the visceral peritoneum, including acellular mucin or mucinous epithelium involving the serosa of the appendix. T4b directly involves adjacent organs or structures, including acellular mucin or mucinous epithelium (1,2,4). 

**Table 1:** Salient Features of Five LAMN Cases

Case	Age/Sex	Gross Findings	Microscopic findings	Pathological Staging	Grade	Complication
1	40/F	Multiple mucinous vesicles on omentum, peritoneum, uterus and ovaries. Appendix: mucin-filled lumen. (Figure 2, 3A&B)	Ovaries: pseudo-stratified tall columnar mucinous epithelium with goblet cells and apical mucin. Appendix: villous architecture with extracellular mucin pools and glands with extension into adjacent peritoneum. (Figure 4,5)	pT4bNxM1b	G1	Pseudomyxoma peritonei, ovarian and peritoneal involvement
2	60/F	Perforated appendix tip and mucinous material in peritoneum	Flattened epithelial growth with pushing invasion and dissection of acellular mucin on the wall. Peritoneal wall: Acellular mucin and inflammatory reaction.	pT4a M1a	G1	Pseudomyxoma peritonei, peritoneal involvement
3	26/F	Previous appendicectomy done for LAMN followed by Hemicolectomy (revised surgery): grey-black area in ileum, mucinous deposits on terminal ileum.	No malignant cells were seen in the mucinous deposits (Revised surgery)	pT3Nx (Previous surgery)	G1	Intestinal obstruction
4	68/F	Dilated, inflamed appendix with mucinous content and peritoneal extension. (Figure 10- B,C)	Appendix: mucinous epithelium, enclosing abundant mucinous material and dissecting into the muscularis propria and serosa. (Figure 11 A,B) Peritoneum: Mucinous deposits present.	pT4a NM1b	G1	Pseudomyxoma peritonei
5	35/M	Enlarged, dilated appendix	mucinous columnar epithelium with abundant mucin in the lumen.	pTisNM	G1	Localized, no peritoneal spread

**Case 1:** Our first case involved a female patient with both appendiceal and ovarian mucinous involvement, ultimately diagnosed as LAMN (G1, pT4b NxM1b). Similar cases have been reported where distinguishing between primary ovarian and appendiceal origin is challenging. Immunohistochemistry (CK20 positivity, CK7 negativity) was crucial in confirming appendiceal origin, consistent with findings by Perivoliotis et al. (1,2,3). and Mohammed N AlAli et al. (13). The presence of PMP and ovarian involvement aligns with studies indicating that synchronous ovarian and appendiceal mucinous tumors are most often metastatic from the appendix.

**Case 2: **This patient presented with perforated appendicitis and mucinous deposits on the peritoneum, staged as pT4a M1a (G1). Literature supports that perforation increases the risk of PMP, even in the absence of overt malignancy. Our case mirrors report by Wang et al. (2), where early surgical intervention and thorough histopathological evaluation were pivotal for management.

**Case 3:** Despite initial management with appendectomy and peritoneal lavage, this young female patient developed intestinal obstruction with mucinous peritoneal deposits, necessitating a right hemicolectomy. This case underscores the potentially aggressive course of pseudomyxoma peritonei (PMP) and highlights the need for close and ongoing surveillance, as emphasized by Misdraji et al. (6) and Kang et al. (9). Notably, the absence of malignant epithelial cells in the mucinous deposits suggests a more favorable prognosis, aligning with observations reported by Lu et al. (4).

**Case 4:** This elderly female presented with acute intestinal obstruction and was found to have LAMN with peritoneal infiltration (pT4a NM1b). The literature documents that advanced-stage LAMN can present with bowel obstruction and extensive peritoneal disease. Misdraji et al. (6) have described that LAMN with serosal penetration or rupture frequently leads to mucinous dissemination in the peritoneal cavity, manifesting as intestinal obstruction or even acute abdomen, especially when mucin accumulates in dependent areas. Kang et al. (9) and Akay et al. (8) further report that peritoneal involvement by LAMN often results in non-specific symptoms such as abdominal pain, distension, and obstruction, and these cases are at high risk for developing pseudomyxoma peritonei. Our findings reinforce the need for prompt diagnosis and surgical management to prevent further morbidity, as highlighted in these studies.

**Case 5:** Unlike the previous cases, this male patient had LAMN confined to the appendix, with no evidence of peritoneal spread. Early detection and management likely prevented progression to PMP, as supported by Akay et al. (8) and Salapathi et al. (12), who found that complete excision of localized LAMN offers an excellent prognosis.

Across our case series, PMP was the most frequent and severe complication, observed in four out of five cases (Table 1). This is in line with published data indicating that mucin spillage, either spontaneous or iatrogenic, is the primary driver of PMP development. The role of immunohistochemistry and tumor markers (CEA, CA19-9, CA125) in differentiating primary sites and predicting recurrence is increasingly recognized, though not yet definitive.

The grading and staging of LAMN are critical for prognosis and management. According to the AJCC and PSOGI criteria, low-grade (G1) tumors confined to the appendix (pTis) have an excellent prognosis, with appendectomy often curative. However, higher-stage disease (pT4a/b) and extra-appendiceal spread, as seen in our series, necessitate more aggressive interventions, including cytoreductive surgery and HIPEC, as advocated by Davison et al. (10) and Kang et al. (9).

Our findings are consistent with several published case series, which report a spectrum of presentations from incidental appendiceal lesions to advanced PMP. These findings are in line with recent case series, including Papatheodorou et al., who highlight the variable presentation of LAMN and the critical role of comprehensive pathological and multidisciplinary evaluation in optimizing patient outcomes (14). The proportion of cases with peritoneal involvement in our series (80%) is slightly higher than some reports, possibly reflecting referral bias or increased awareness at our institution.

Among the different cases presented above, patients presenting with early symptoms of acute appendicitis need to be looked into carefully because patients with later symptoms of intestinal obstruction must raise a suspicion of pseudomyxoma peritonei.

Mucinous adenocarcinoma is defined by infiltrative destructive invasion and high-grade cytologic features (present at least focally) and may have areas of both low and high cytologic grades (6,8). It may also have a signet ring cell component. PSOGI and AJCC 8th edition advocate a three-tier grading system for mucinous neoplasia: G1, well-differentiated (low cytologic grade, corresponding to LAMN); G2, moderately differentiated (high cytologic grade without signet ring cells); G3, poorly differentiated (high cytologic grade, usually with signet ring cells).

For the purpose of therapeutic decision-making, a two-tier grading system is often used. Patients with disseminated low-grade (G1) disease benefit from cytoreductive surgery with hyperthermic intraperitoneal chemotherapy (CRS-HIPEC), with no role for systemic chemotherapy. Patients with disseminated high-grade (G2 and G3) disease are often treated with systemic chemotherapy, with the option of CRS-HIPEC at some institutions (8,10). The role of CRS-HIPEC is not entirely well delineated, although it is used aggressively at many centers with evidence of survival benefit (3,5).

On the prognosis front, LAMN or mucinous adenocarcinoma has a 10-year survival rate (10,11). However, prognosis also depends on the tumor grade and underlying molecular alterations, such as mutations in KRAS, BRAF, and GNAS. The estimated 10-year survival rates for G1, G2, and G3 tumors are approximately 50%, 30%, and 10–20%, respectively (10, 11).

Postoperatively, it is advisable to monitor for metastasis following appendectomy in patients with LAMN, as mucinous material containing malignant cells may be spilled into the peritoneal cavity during surgical removal. Therefore, a contrast-enhanced CT (CECT) or PET scan should be considered during follow-up to help prevent complications such as pseudomyxoma peritonei. 

## Conclusion

LAMNs confined to the appendix are rare and should be differentiated from serrated lesions and diverticula. Grading and staging of mucinous neoplasm of appendix and Pseudomyxoma Peritonei needs a unified approach for standardized diagnostic reporting. Extra-appendiceal mucin is important for staging and prognosis. Thus, a ruptured or perforated appendix must warrant for an immediate suspicion of pseudomyxoma peritonei which when untreated can lead to mucinous neoplasms in adjacent organs like ovary and colon. Pseudomyxoma Peritonei with simultaneous appendix and ovarian neoplasm should be treated as primary appendiceal tumour. 

## Data Availability

Data is available upon reasonable request from the corresponding author.
